# Gold-based nanoparticles realize photothermal and photodynamic synergistic treatment of liver cancer and improve the anaerobic tumor microenvironment under near-infrared light

**DOI:** 10.3389/fbioe.2022.957349

**Published:** 2022-08-17

**Authors:** Bei Li, Yi Fu, Maodi Xie, Lei Feng, Xiaoya Niu, Lin Que, Zhen You

**Affiliations:** ^1^ Division of Biliary Surgery, Department of General Surgery, West China Hospital, Sichuan University, Chengdu, China; ^2^ Research Center for Biliary Diseases, West China Hospital, Sichuan University, Chengdu, China; ^3^ Department of Physiology and Pathophysiology, School of Basic Medical Sciences, Peking University, Beijing, China; ^4^ West Chia-Washington Mitochondria and Metabolism Center, West China Hospital of Sichuan University, Chengdu, China; ^5^ Department of Head and Neck Oncology, West China Hospital of Stomatology, Sichuan University, Chengdu, China; ^6^ State Key Laboratory of Oral Diseases, West China College of Stomatology, Sichuan University, Chengdu, China

**Keywords:** chlorin e6, liver cancer treatment, photodynamic therapy, photothermal therapy, gold nanoparticles

## Abstract

In order to solve the different pains caused by traditional cancer treatment methods such as surgical treatment, the nano-drug delivery system provides new ideas for cancer treatment. In this paper, a novel anti-tumor therapy nanoparticle, P(AAm-co-AN)-AuNRs@CeO_2_-Ce6(PA/Ce6), is prepared, which provides a novel idea for liver cancer treatment. The CeO_2_-coated gold nanorods were grafted onto the surface of the temperature-sensitive polymer P(AAm-co-AN)-CTPD. The photosensitizer Ce6 is loaded on the surface of the nanoparticles and the polymer layer. CeO_2_ can effectively alleviate the tumor anaerobic microenvironment, and under 808 nm near-infrared (NIR) excitation, the gold nanorods achieve photothermal conversion to induce local heating, which leads to the phase transition of the polymer layer and realizes a controllable release mechanism. In addition, 660 nm NIR light can effectively induce Ce6 to produce singlet oxygen, thereby effectively killing cancer cells. Under the 808 nm laser irradiation within 600 s, the PA/Ce6 solution can heat up to about 60°C, which was enough to ablate both cancer cells and tumor tissues. When the temperature was 50°C, the cumulative release rate of Ce6 was 95.31%. Under the 808 nm laser irradiation, oxygen production capacity of PA/Ce6 was higher and can effectively reduce the content of hydrogen peroxide in cancer cells. Compared to free Ce6, the reactive oxygen species-mediated fluorescence of PA/Ce6 nanoparticles was greater. The cell viability and migration of HepG2 cells were decreased after the 660 and 880 nm lasers were irradiated at the same time. The cancer cells were further inhibited, showing a good *in vitro* anti-tumor effect. PA-DOX showed the best tumor growth inhibitory effect under NIR laser irradiation and had no acute toxicity *in vivo*. Due to the existence of AuNRs, nanoparticles had high-efficiency photothermal conversion ability to achieve photothermal therapy. Ce6 can generate singlet oxygen under the excitation of 660 nm laser to realize photodynamic therapy. The experimental results also showed that PA/Ce6 can effectively decompose hydrogen peroxide under laser irradiation, aiming to effectively alleviate the anaerobic microenvironment of tumors. These indicate that PA/Ce6 plays a promising role for hepatocellular carcinoma treatment.

## Introduction

As one of the most frequent malignancy worldwide, hepatocellular carcinoma (HCC) is expected to have a further increasing incidence rate in the next few years in Southeast Asia and Africa due to the increase in hepatitis B and C virus infections (Rashed et al., 2020; Shen et al., 2020; Lee et al., 2021; McGlynn et al., 2021). At present, although traditional comprehensive treatment methods such as surgery, interventional therapy, molecular targeted therapy, and radiotherapy play a crucial role in HCC systemic treatment, the therapy results still remain limited (Chen and Wang, 2015; Zhu et al., 2019). Certain methods that targeted therapy and radiotherapy can not only prolong HCC patients’ overall survival but also often cause severe toxicity and drug resistance to the body (Zhou and Song, 2021).

Studies have shown that the capillary structure in tumor tissues is not perfect and presents a porous structure (the pore size is about tens to hundreds of nanometers) and has super-permeability (Sarin, 2010; Manzano and Vallet-Regí, 2020). When the nanoparticles in the blood flow through the capillaries, they can leak out from the nanopores and enter the tumor tissues to accumulate in this tissue [enhanced permeability and retention (EPR) effect] (Shi et al., 2020). Therefore, the longer the drug delivery system is in the blood circulatory system, the more times it flows through the tumor capillaries and the accumulation in the tumor. In recent years, traditional anti-tumor drugs have been loaded in nano-carriers for the purpose of enhancing anti-tumor drug water solubility and passively targeting tumor tissues to improve the therapeutic effect (Fang et al., 2020). The concentration of the drug in healthy tissues reduces to avoid the side effect, while the anti-tumor effect is not compromised. In addition, “smart” nano-delivery particles, such as temperature-sensitive or pH-responsive nanoparticles, become one of the research hotspots in cancer therapy. Among them, P(AAm-co-AN), as an acrylamide–acrylonitrile copolymer, can adjust its response temperature *via* regulating the feed ratio of acrylonitrile (AN) and anthranilamide (AAm). It has been widely used in the development and application of nano-drug carriers (Zhang et al., 2014; Hou and Wu, 2015). A previous study has shown that among the physical properties of AuNPs, localized surface plasmon resonance (LSPR), radioactivity, and a high X-ray absorption coefficient are widely used in the diagnosis and treatment of tumors. As an advantage over many other nanoparticles in chemicals, AuNPs can form stable chemical bonds with S-and N-containing groups. This allows AuNPs to attach to a wide variety of organic ligands or polymers with a specific function. These surface modifications endow AuNPs with outstanding biocompatibility, targeting, and drug delivery capabilities (Bai et al., 2020). The gold-core silica shell (AuMSS) nanoparticles’ advantageous physicochemical and biological properties make them promising nanoplatforms for cancer therapy. The successful modification of the AuMSS nanospheres with PEOZ and β-CD as well as their promising properties are suitable for application in cancer therapy (Reis et al., 2019).

Photodynamic therapy (PDT), one regular method for cancer treatment, uses a photosensitizer (PS) to touch off a battery of photobiological and photochemical reactions under laser shining of a specific wavelength (Dang et al., 2017; Hwang et al., 2018; Juarranz et al., 2020). The most important thing is that it produces singlet oxygen to trigger tumor cell release with thromboxanes, lymphokines, prostaglandins, and other cytokines to kill tumor cells by destroying the micro-vessels of tumor cell biofilms and tissues. Animal experiments and clinical applications have found good curative effects (Moret and Reddi, 2017). Therefore, the development of photosensitizers has become a focus and hot spot in PDT treatment. Chlorin e6 (Ce6) is usually synthesized from pheophorbide a. It is a good photosensitizer with a biological activity similar to that of pheophorbide a. Ce6 has a high productive efficiency for singlet oxygen, which is fit for PDT development for tumors (Beack et al., 2015; Fu et al., 2020). However, like most photosensitizers so far, Ce6 is hydrophobic and easily aggregates in solution, so it has certain difficulties in practical applications.

In addition, PDT that kills cancer cells by using reactive oxygen species has been widely used for cancer therapy. Nevertheless, the PDT efficiency is badly limited by the anoxic characteristics of most solid tumors. Hypoxia occurs due to abnormal growth of tumor cells and dysfunction of the vasculature (Zou et al., 2016). During hypoxia, some unwanted metabolites are produced, for example, hydrogen peroxide (H_2_O_2_). These metabolites can advance cancer cell metastasis and mutagenesis. At the same time, hypoxia can also cause tumor resistance to PDT, leading to treatment failure. In recent years, photothermal therapy (PTT) has developed into an alternative or adjuvant therapy for cancer treatment. PTT is a non-invasive tumor treatment method that uses photothermal materials to convert light energy into local overheating, thereby killing cancer cells (Liu et al., 2019; Liu et al., 2020). In addition, it has the advantages of non-invasiveness, precise controllability, and so on. PTT can not only promote the ablation of cancer cells but also induce local vascular damage, increase the blood perfusion of tumor tissues, and improve the hypoxic state inside the tumor. The 700–1,400 nm near-infrared (NIR) laser used in the treatment technology has a very strong ability to penetrate biological tissues, and the light absorption attenuation during the penetration process is small (Ke et al., 2020). A prerequisite for the effective use of NIR PTT is the development of efficient, biocompatible, and targeted NIR photothermal conversion reagents. As one of the typical photothermal reagents, gold nanorods are characterized by more special surface plasmon resonance (SPR) compared with spherical gold nanoparticles: there are two SPR peaks in the horizontal and vertical directions. The SPR peak value on the vertical axis (from the visible region to the NIR region) is determined by the major–minor axial ratio of the gold nanorod particles, through controlling which the SPR peak can be adjusted artificially (Ali et al., 2017; An et al., 2019; Reed et al., 2019). Therefore, the gold nanorod bioprobe can match the excitation wavelength of the NIR, which can make the NIR light penetrate the deep subcutaneous tissue and use the gold nanorod under the ultrashort pulse infrared laser to realize the imaging and photothermal features of the deep subcutaneous tissue treatment. Therefore, the combination of hyperthermia and PDT may cause a synergistic anti-tumor response (Huang et al., 2019).

As a rare earth material, cerium dioxide (CeO_2_) has excellent catalytic properties due to the reversible conversion of cerium ions between Ce^3+^ and Ce^4+^ and the presence of oxygen vacancies. Studies have shown that CeO_2_ nanoparticles have superoxide dismutase (SOD), catalase (CAT), and oxidase-like activities and can also scavenge hydroxyl radicals and nitric oxide free radicals (Asadujjaman et al., 2017).

Based on the above discussion, we designed a novel kind of composite nanoparticles for HCC treatment. The CeO_2_-coated gold nanorods were grafted onto the surface of the temperature-sensitive polymer P(AAm-co-AN)-CTPD, and it was used at low temperature. The photosensitizer Ce6 is loaded on the surface of the nanoparticles and the polymer layer. CeO_2_ can effectively alleviate the tumor anaerobic microenvironment, and under 808 nm NIR excitation, the gold nanorods achieve photothermal conversion to induce local heating, which leads to the phase transition of the polymer layer and realizes a controllable release mechanism. In addition, 660 nm NIR light can effectively induce Ce6 to produce singlet oxygen, thereby effectively killing cancer cells and achieving anti-tumor properties.

## Methods

### Materials

Ce6 from Bai Lingwei Technology Co., Ltd. (Shanghai, China) was obtained; l-ascorbic acid (AA), isopropylamine, and cetyltrimethylammonium bromide (CTAB) were obtained from Sigma-Aldrich (Missouri, United States); acrylonitrile (AN) was obtained from Aladdin Industrial Inc. (Shanghai, China); silver nitrate (AgNO_3_) and hydrogen tetrachloroaurate (III) hydrate (HAuCl_4_.4H_2_O) were obtained from Macklin Reagent (Shanghai, China); all other solvents and reagents were obtained from Sinopharm Chemical Reagent Inc. (Shanghai, China).

### Cells and animals

In the experiments, HepG2 cells and female nude mice (weighing 20 ± 2 g) were provided by Nanjing KeyGen Biotechnology and Qinglongshan Animal Farm (Nanjing, China), respectively. The experiments were approved by the Animal Ethics Committee of West China Hospital of Sichuan University, strictly conforming to the ethical code and the Regulations of the People’s Republic of China on the Administration of Laboratory Animals.

### Synthesis of P(AAm-co-AN)-CPTD

First, AAm (1.452 g), 10 ml of dioxane was used to dissolve AN (0.192 g), azodiisobutyronitrile (AIBN) (0.0196 g), and CPTD (0.16 g) in a 50 ml three-necked flask. The mixture was thoroughly deoxygenated by purging N_2_ and tertiary freeze-pump-thaw cycles and then continually reacted at 65°C for 24 h, followed by rapid cooling with ice water. The mixture was purified more than three times in ice methanol to remove impurities and unreacted monomers. The final product was obtained as P(AAm-co-AN)-CPTD. A small amount of the sample was dissolved in CDCl_3_, and ^1^H NMR was applied to analyze its chemical structure.

### Synthesis of AuNRs

5 ml of 0.1 M CTAB solution (containing 13.5 µl of isopropylamine) was added to a 25 ml beaker; then, 500 µl of 0.5 mm HAuCl_4_ solution, 32.5 µl of 40 mm AgNO_3_ solution, and 50 µl of hydroxylamine hydrochloride solution were added separately. Afterward, the mixture was continuously stirred and 6 µl of NaBH_4_ solution was added. This mixture was transferred to a water bath at 30°C for 11 min. While stirring, 5 ml of 200 mm CTAB solution, 5 ml of deionized (DI) water, 32.5 µl of 40 mm AgNO_3_ solution, and 5 µl of 9.29 M NiSO_4_ solution were added, and finally, the mixture was transferred to a cooling cycle at 18°C for 24 h. The AuNRs were purified by high-speed centrifugation, dispersed in DI water, and then kept refrigerated.

### Synthesis of AuNRs@CeO_2_

10 ml of the AuNR solution was re-dispersed in 8 ml of 0.025 M CTAB solution after high-speed centrifugation. After that, 0.8 ml of 0.1 M EDTA-NH_3_ solution and 80 µl of 0.1 M Ce(NO_3_)_3_ solution were added to it. The mixture was shaken for 2 min and transferred to an oil bath at 90°C to react for 5–6 h. Then, the AuNRs coated with CeO_2_ were obtained by centrifugation at 5,500 rpm for 30 min.

### Synthesis of PA/Ce6

10 mg of P(AAm-Co-AN)-CPTD and 2 mg of Ce6 were dissolved in 2 ml of the mixed solvent of tetrahydrofuran and dimethylformamide (2:3, volume/volume). Under magnetic stirring, the mixed solution was added dropwise to the AuNRs@CeO_2_ solution using a syringe pump at 10 ml/h rate, maintained for 24 h, and dialyzed for 24 h with DI water to remove the organic solvent to obtain PA/Ce6. After that, we verified whether Ce6 is present in the PA/Ce6 nanocomplexes *via* a UV−vis spectrophotometer (Lengguang Technology, Shanghai). We measured the size/surface potential and morphology of PA/Ce6 nanocomplexes by dynamic light scattering (DLS) (Brookhaven Instruments, United States) and transmission electron microscopy (TEM) (JEM-2100 JEOL, Japan), respectively. We calculated the Ce6 encapsulation efficiency (EE) and loading efficiency (LE) with Eqs. 1, 2:

EE =(weight of total encapsulated of Ce6)/(total feeding weight of Ce6) ×100% (1)

LE =(weight of Ce6 in the nanocomplexes)/(weight of nanocomplexes)×100% (2)

### Characterization

AuNRs and PA/Ce6 were characterized by TEM. AuNRs, AuNRs@CeO2, and PA/Ce6 were characterized by DLS. AuNRs, AuNRs@ceO2, Ce6, and PA/Ce6 were characterized by ultraviolet spectroscopy (UV–vis). P(AAm-co-AN)-CPTD was characterized by nuclear magnetic resonance (1H NMR). AuNRs, AuNRs@CeO2, and PA/Ce6 were characterized by zeta potential (ZP).

### Photothermal performance studies

The photothermal performance of PA/Ce6 was studied using an 808 and 660 nm semiconductor laser device (LE-LS-808-XXTM/LE-LS-660-XXTM, Leoptics, Shenzhen) with different irradiation powers. Irradiation was performed with a Ga-Al-As diode laser (Photon Laser III, DMC^®^), a continuous mode, and wavelengths (*λ*) of 660 and 808 nm at different energy densities (EDs) (j/cm^2^) and power densities (PDs) (W/cm2). Laser irradiation was performed 20 min after placing LPS in the wells. For each power setting (P) of 50 mW, an exposure time (T) of 30 s (s) was used in the two wavelengths. We stimulated the pa/ce6 aqueous solution and recorded the real-time temperature every 10 s with an electronic thermometer. Phosphate-buffered saline (PBS) was used as the control group. We calculated the PA/Ce6 thermal conversion efficiency based on the response curves according to Eq. 3

$$\text{η}=\left[\text{h∗s}\left({\text{T}}_{\text{max}}\text{,}\text{NP}-{\text{T}}_{\text{surr}}\right)-{\text{Q}}_{\text{dis}}\right]\text{/I}\left({1-10}^{-\text{A}808}\right)$$ (3)

where s is the container surface area, T_max_ is the thermal peak of the solution, h is the parameter of heat conductivity, I is the laser intensity, T_surr_ is the surrounding temperature, Q_dis_ is the heat produced when water and the container absorb light, and A^808^ is the absorption value of the material at 808 nm. For calculation of h*s, we use Eqs. 4, 5

$${\text{Q}}_{\text{dis}}=\text{h∗s}\left({\text{T}}_{\text{max}\text{,}\text{H}2\text{O}}-{\text{T}}_{\text{surr}}\right)$$ (4)

$$\text{τs}=\left({\text{m}}_{\text{D}}{-\text{c}}_{\text{D}}\right)\text{/}\left(\text{h∗s}\right)$$ (5)

where m_D_ means the water quality; c_D_ means the water heat capacity (4.2 J/g/°C); and τs is the time constant of the sample system, calculated with Eqs. 6, 7

t=-τsln⁡θ (6)

$$\theta =\left({\text{T}}_{\text{surr}}-T\right)/\left({\text{T}}_{\text{surr}}-{\text{T}}_{\text{max}}\right)$$ (7)

### Ce6 release from nanoparticles

We packed 10 mg of PA/Ce6 in a dialysis bag [molecular weight cutoff (MWCO) = 3.5 kDa], immersed it in PBS buffer, and incubated in the dark at 4°C, 37°C, and 50°C for a pre-specified time. We extracted 3 ml of the solution and replenished with 3 ml of the fresh buffer in time. The absorption value was gauged in an ultraviolet–visible spectrophotometer, and the concentration of Ce6 was calculated. The experiment was carried out in replicate.

### H_2_O_2_ decomposition and O_2_ generation assay

The ^1^O_2_ generated by PA/Ce6 under NIR laser (660 and 808 nm) irradiation was studied by the spectroscopic method based on 1,3-diphenylisobenzofuran (DPBF). In short, the PA/Ce6 or PBS solution (3 ml) was added with N, N-dimethylformamide solution (20 µL) which contained DPBF at 1 mg/ml, placed in the dark, and laser-irradiated at 660 nm for different times. After centrifugation, we recorded the supernatant’s absorbance at 410 nm using an ultraviolet–visible spectrophotometer. Free Ce6 was used as the control group.

100 µM H_2_O_2_ and 200 μg/ml Au@CeO_2_ or PA/Ce6 and 15 ml of PBS were uniformly blended in a centrifuge tube. The mixture solution was treated with or without 808 nm laser irradiation. The solution absorbance at 405 nm was measured every 10 min by using a hydrogen peroxide determination kit to evaluate the H_2_O_2_ concentration. In addition, the level of O_2_ production was monitored every 10 min with a portable dissolved oxygen meter.

### *In vitro* cell uptake assay

Cell uptake for these nanoparticles was qualitatively analyzed by confocal laser scanning microscopy (CLSM). First, we cultured HepG2 cells for 48 h and inoculated to a 12-well plate at 1×10^5^ cells/well. Then, we rinsed the cells with a fresh medium and co-cultured them with the nanoparticles for 4 h. We incubated HepG2 cells with 50 mm H_2_O_2_ (200 µL) at 37°C for 30 min as a positive control. Afterward, we fostered the cells for 1 h with 10 mm H_2_DCFDA (50 µL) at 37°C, followed by three times washing with PBS and 5 min exposure to laser shining (660 nm, 0.2 W/cm^2^). Finally, we observed the cells by CLSM.

Intracellular H_2_O_2_ assay was applied to evaluate the intracellular H_2_O_2_ concentration. First, HepG2 cells were added with 100 µm H_2_O_2_ solution and pre-incubated for 24 h with 200 μg/ml Au@CeO_2_ and PA/Ce6. Subsequently, the mixture was laser-irradiated for 5 min (at 808 nm) and then incubated for 3 h. Afterward, we replaced the culture medium with PBS and measured the H_2_O_2_ concentration in the cells with a fluorescent hydrogen peroxide detection kit (Sigma-Aldrich, ex/em = 490/520 nm).

### *In vitro* cytotoxicity assay

The standard 3-(4,5-dimethylthiazol-2-yl)-2,5-diphenyltetrazolium bromide (MTT) method was used for detection of nanoparticle cytotoxicity. MTT was reduced by succinate dehydrogenase in the mitochondria into a blue-purple precipitate that is insoluble in water, while dead cells have no such function. The blue-purple precipitate can be dissolved by DMSO, and the absorbance at 490 nm was determined to reflect the living cell number as well as its proliferation. In the experiment, 96-well plates were used to foster different concentrations of the nanoparticle suspension and HepG2 for 24 h. Only nanoparticle suspensions were added to the control group. Then, each well was supplied with MTT solution (20 μl, 5 mg/ml) and incubated for 4 h. Finally, we aspirated the medium out and added 150 μl of DMSO, followed by testing the absorbance value for each well.

### *In vitro* cellular migration assay

A fetal bovine serum (FBS)-free fresh culture medium was co-cultured with HepG2 cells in logarithmic growth for 12 h. The cells were starved, collected, and resuspended in different components of the FBS-free fresh culture medium (AuNRs, AuNRs@CeO_2_, free Ce6, and PA/Ce6). 100 µl of HepG2 cells with different treatments was added to a 24-well transwell chamber (pore size: 8 μm, Corning); the number of cells was 1×10^5^, and the fresh culture solution supplementary in 10% FBS was added to the 24-well culture plate as a chemical attractant. After that, the plate was kept for 48 h in a CO_2_ incubator at 37°C. With the help of a cotton swab, we carefully removed the remaining non-migrated or invaded cells; we fixed invaded or migrated cells that adhered to the lower surface of the transwell chamber with 4% paraformaldehyde for 20 min and stained them for 10 min with 0.5% crystal violet solution. Cell migrations in multiple fields of view were observed, photographed, and recorded with an inverted microscope.

### *In vivo* anti-tumor efficacy assay

A total of 2×10^6^ HepG2 tumor cells were injected subcutaneously into mice to establish a subcutaneous liver cancer tumor model. By the time the tumor grew to 200 mm^3^, the nude mice were divided into six groups randomly (*n* = 6 for each group) [Saline, Saline + light (808 nm + 660 nm laser), PA/Ce6, PA/Ce6 + 808 nm laser, PA/Ce6 + 660 nm laser, and PA/ce6+light (808 nm + 660 nm laser)]. After that, tail vein injection of nanoparticles was conducted on mice. 12 h later, the tumors were laser-irradiated locally. We monitored the mice weight and tumor volume every other day and euthanized the mice on the 15th day after the treatment. The tumors were taken out to take pictures.

### Data analysis

Statistical analysis was performed using the “R” software package (Version 4.1.1) and GraphPad Prism verson 9.0 (GraphPad software, San Diego, CA). Data were analyzed with the help of GraphPad Prism 9.0 software and based on the one-way ANOVA test or two-way ANOVA test. All statistics were presented as mean ± standard deviation (SD). * indicates a *p* value of <0.05, and ** indicates a *p* value of <0.01.

## Results and discussion

### PA/Ce6 characterization

**FIGURE 1:**
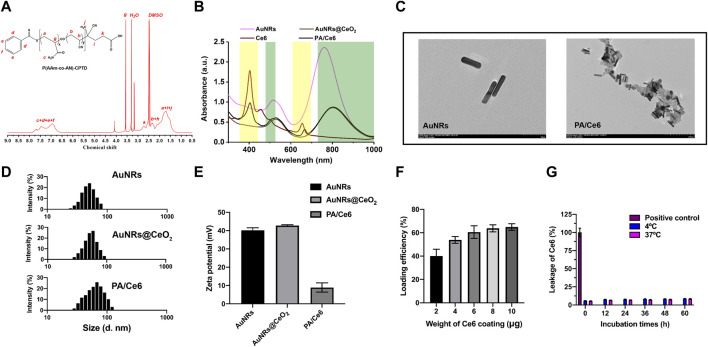
**(A)** ^1^H-NMR spectra of P(AAm-co-AN)-CPTD in DMSO-*d*_6_, **(B)** UV–vis absorption spectra of AuNRs, AuNRs@ceO2, Ce6, and PA/Ce6, **(D)** TEM image of AuNRs and PA/Ce6, **(C)** DLS image of AuNRs, AuNRs@CeO2, and PA/Ce6, **(D)** zeta potential of AuNRs, AuNRs@CeO2, and PA/Ce6, **(F)** loading efficiency of Ce6 in PA/Ce6, and **(G)** leakage of Ce6 in PA/Ce6. For each experiment, three independent repetitions were conducted in this study. * indicates a *p* value of <0.05; ** indicates a *p* value of <0.01.

In this research, CPTD acted as a chain-transfer agent for preparation of UCST polymer P(AAm-co-AN)-CPTD through a typical RAFT reaction. The P(AAm-co-AN)-CPTD chemical structure was analyzed through ^1^H NMR (see Figure 1A). The signal at 6.5–7.5 ppm proves the existence of the amino proton unit [31], while the signal at 1.2–2.4 ppm is attributed to the -CH_2_-CH- proton on P(AAm-co-AN)-CPTD [32]. The UV-vis-NIR absorbance spectra of AuNRs, AuNRs@CeO_2_, Ce6, and PA/Ce6 were obtained using an ultraviolet spectrophotometer as shown in Figure 1B. AuNRs showed two absorption peaks at 500 and 780 nm, corresponding to the transverse and longitudinal SPR peaks, respectively. When coated with CeO_2_, the longitudinal peaks of AuNRs (located at 808 nm) showed a significant red shift. In addition, it can be seen that the characteristic peaks of Ce6 appeared in the PA/Ce6 absorption curve, indicating the successful loading of the photosensitizer. TEM was utilized to analyze the PA/Ce6 morphology. As demonstrated in Figure 1C, the pristine AuNRs presented a rod-like structure with a smooth surface and an aspect ratio of about 4:1. After the coating of CeO_2_, we clearly saw a rough layer on the surface of PA/Ce6, confirming the coating of CeO_2_. The zeta potential and DLS analysis were also used to monitor the changes after the CeO_2_ coating and Ce6 loading. As illustrated in Figure 1E, before the deposition of CeO_2_, AuNRs showed a positive surface charge of about 40 mV. The coating of CeO_2_ did not markedly affect the zeta potential. However, the zeta potential was greatly suppressed after Ce6 loading. The decrease of the potential value of PA/Ce6 indicated the successful loading of Ce6. In addition, it can be expected that the size of PA/Ce6 nanoparticles increased significantly compared with AuNRs. The average hydrodynamic diameter of AuNRs@CeO_2_ was 55 nm. For PA/ce6, the average size increased to 70 nm with a polydispersity index around 0.109.

For Ce6 loading, Ce6 in different amounts was fostered with nanoparticles, and then free Ce6 was removed after multiple centrifugation. To obtain PA/Ce6 with a high CE6 load, the addition amount of CE6 can be increased (>60%) (Figure 1F). Compared with free Ce6, the leakages of PA/Ce6 nanoparticles after 72 h of incubation in DI H_2_O at 4 and 37°C were all less than 10%, indicating that the package of Ce6 in the nanoparticles was stable (Figure 1G).

### Photothermal performance

**FIGURE 2:**
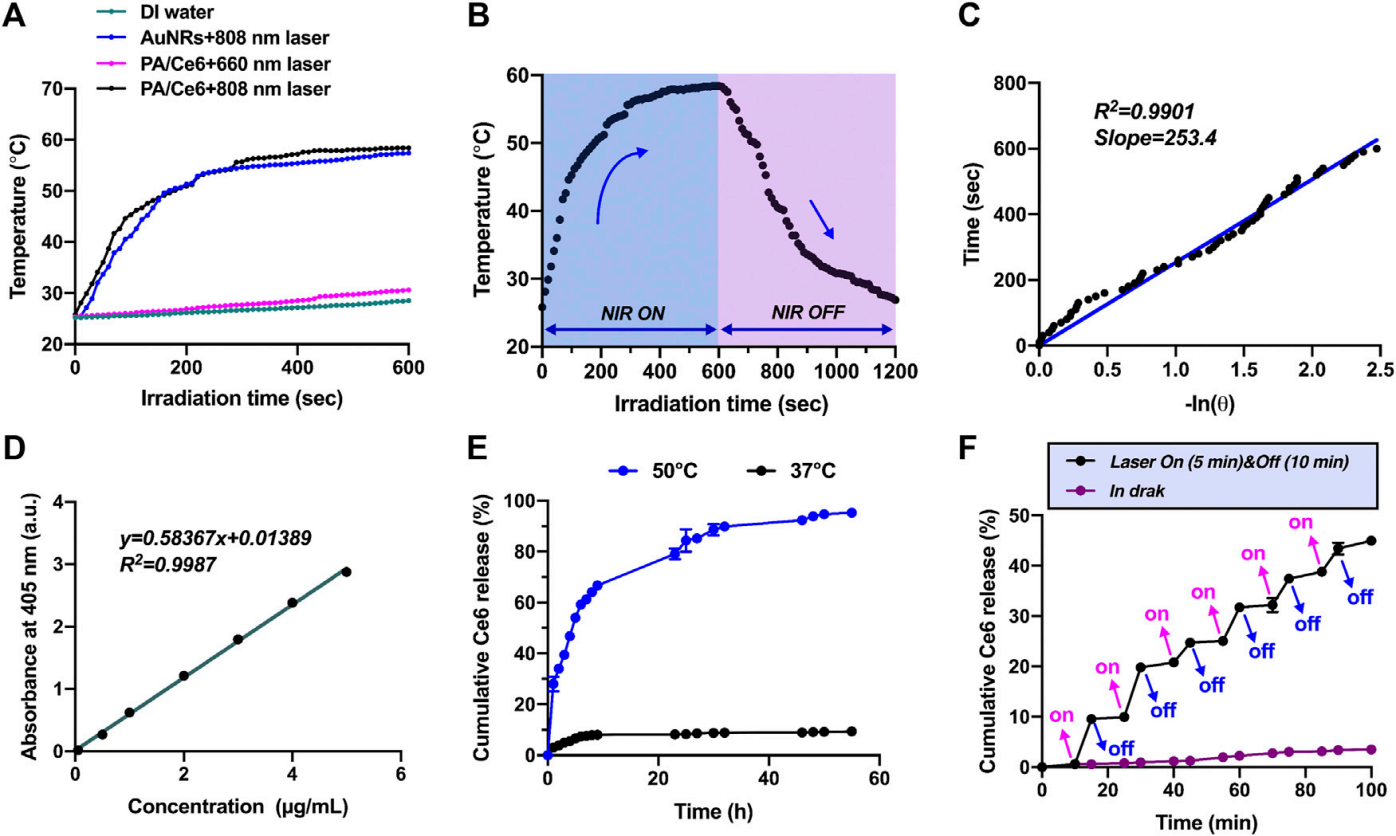
**(A)** Temperature change curves of PBS, AuNRs, and PA/Ce6 solution under NIR laser irradiation at 860 and 660 nm, **(B)** temperature curves of PA/Ce6 solution in one single on/off process of NIR laser irradiation, **(C)** association of cooling time with the negative natural logarithm of temperature driving force after turning off NIR laser, **(D)** standard solution curve of Ce6, **(E)** cumulative Ce6 release curves of PA/Ce6 in PBS (pH 7.4) at 37°C and 50°C, and **(F)** Ce6 release curve of PA/Ce6 over 100 min under the cycles of NIR laser on/off. For each experiment, three independent repetitions were conducted in this study. * indicates a *p* value of <0.05; ** indicates a *p* value of <0.01.

The influence of NIR laser irradiation on the photothermal conversion performance of PA/Ce6 was determined. The change of PA/Ce6 nanoparticles with temperature was discovered equivalent to that of AuNRs under 808 nm laser, showing that CeO_2_ coating and Ce6 loading did not alter AuNR photothermal properties (Figure 2A). Under the 808 nm laser irradiation within 600 s, the PA/Ce6 solution can heat up to about 60°C, which was enough to ablate both cancer cells and tumor tissues. Moreover, the experiments also revealed that 606 nm laser irradiation hardly contributed to the remarkable temperature increase to PA/Ce6 (Figure 2A). In Figure 2B, under the condition of 808 nm laser irradiation-off control, the temperature change of the PA/Ce6 nanoparticle solution during the 20 min of heating and cooling was monitored and recorded. The solution can drop from about 60°C to room temperature within 10 min. According to the highly linear relationship curve between the cooling time after NIR laser was turned off and the negative natural logarithm of the temperature driving force (*R* ^2^ = 0.9901), the PA/Ce6 photothermal conversion efficiency was found to be 33.8% (Figure 2).

### Drug loading and release

To monitor the cumulative release of Ce6 from nanoparticles under 808 nm laser irradiation, the release performance at different temperatures (37 and 50°C) was studied. First of all, in order to accurately and conveniently measure the concentration of Ce6, the absorption values of the standard samples at various concentrations were detected using a UV–vis spectrophotometer and the standard solution curve was drawn. The standard curve of Ce6 was constructed with a concentration range of 0–5 mg/ml, showing a linear regression equation of $\text{y}=\text{0}\text{.}58367\text{*x}+\text{0}{\text{.}01389\;(\text{R}}^{2}=\text{0}\text{.}9987)$ (Figure 2D). The 55 h cumulative release curve of Ce6 in nanoparticles at different temperatures is displayed in Figure 2E, indicating that the release ratio of nanoparticles gradually increased within the first 12 h and then leveled off. At 37°C, the cumulative Ce6 release ratio of PA/Ce6 within 55 h reached 9.35%. Correspondingly, when the temperature was 50°C, the cumulative release rate was 95.31%. It demonstrated that temperature indeed had a significant effect on the release behavior of nanoparticles. Compared with the case without NIR irradiation, when NIR irradiation was applied for 5 min, the release pattern suddenly rose (Figure 2F). These results clearly indicated that the release of Ce6 can be increased under the irradiation at specific sites of action. This can be attributed to the increase in the temperature of the nanoparticles induced by laser irradiation, which may trigger the phase transition of the polymer layer on PA/Ce6. The polymer chain stretched, triggering the release of more Ce6.

### Analysis of PA/Ce6 ROS level

**FIGURE 3:**
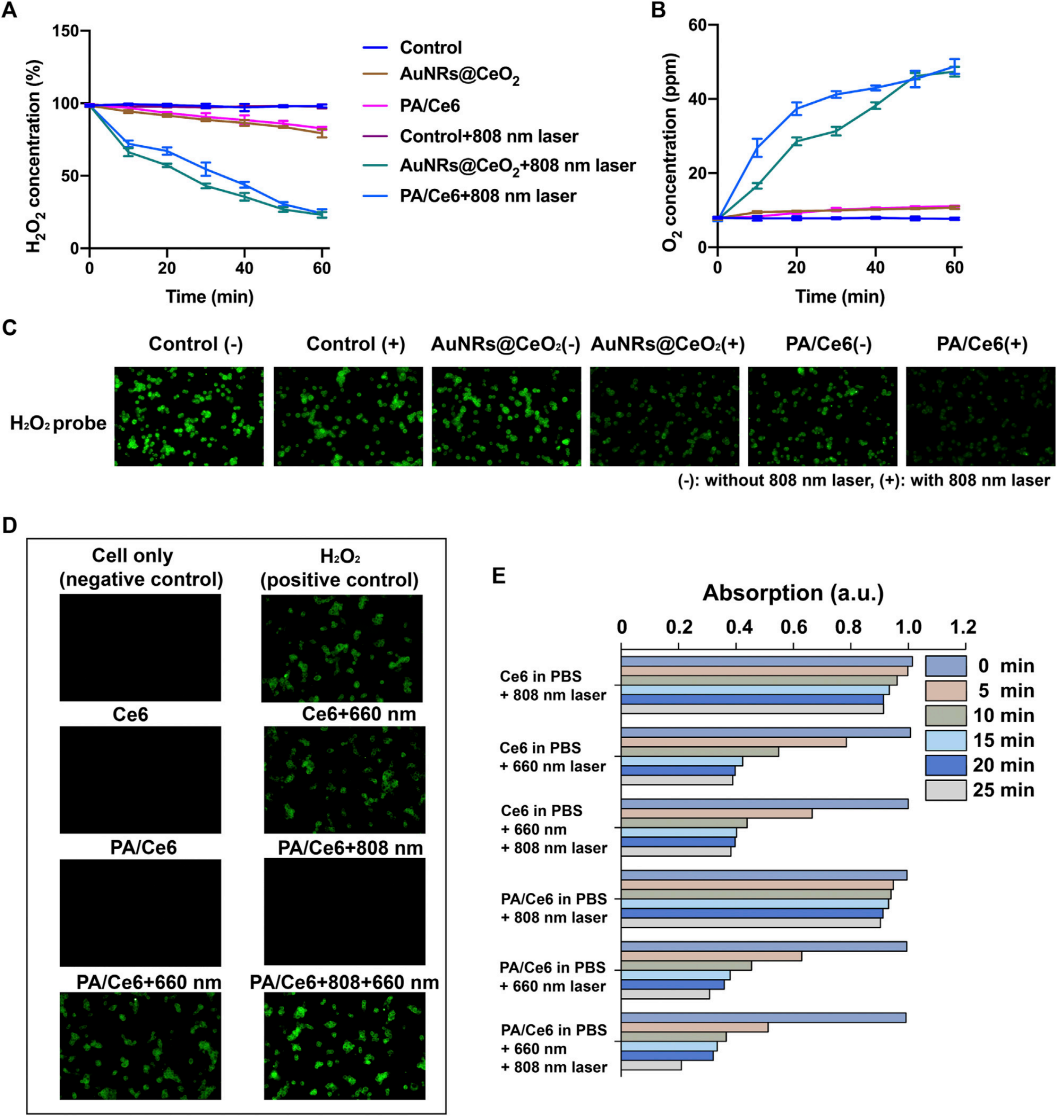
**(A)** H_2_O_2_ concentration in AuNRs@CeO_2_ and PA/Ce6 with/without 808 nm laser rays, **(B)** O_2_ concentration in 100 µm H_2_O_2_ solution over time under different conditions, **(C)** CLSM images of H_2_O_2_ in HepG2 cells treated with AuNRs@CeO_2_ and PA/Ce6 (with/without NIR laser rays) in the presence of H_2_O_2_, **(D)** intracellular ROS generation of PA/Ce6 in HepG2 cells, and **(E)** normalized absorbance of DPBF at 410 nm during photodecomposition by ^1^O_2_ in the presence of Ce6 and PA/Ce6 under different conditions. For each experiment, three independent repetitions were conducted in this study. * indicates a *p* value of <0.05; ** indicates a *p* value of <0.01.

It is well known that previous studies have reported that CeO_2_ can effectively degrade H_2_O_2_ into O_2_ and H_2_O by acting like a hydrogen peroxide mold. Without laser irradiation, the levels of degradation of H_2_O_2_ in each group were equal. However, when irradiated by an 808 nm laser, both PA/Ce6 and AuNRs@CeO_2_ showed a significant ability to degrade H_2_O_2_. PA/Ce6 and AuNRs@CeO_2_ decomposed 80.5 and 81.7% of H_2_O_2_ in 60 min, respectively (Figure 3A). Correspondingly, the oxygen change curve within 60 min also confirmed the oxygen production ability of Au@CeO_2_ and PA/Ce6 under NIR laser (Figure 3B). From the perspective of cancer cells, the effective oxygen production capacity of PA/Ce6 can alleviate the anaerobic microenvironment in cancer cells and effectively improve the negative effects of oxidative stress. In addition, Figure 3C shows the CLSM image of H_2_O_2_ in HepG2 cells treated with AuNRs@CeO_2_ and PA/Ce6 (with/without NIR laser irradiation). The results show that under laser irradiation, PA/Ce6 can effectively reduce the content of hydrogen peroxide in cancer cells.

In order to examine the photodynamic effect, an oxidation-responsive fluorescent probe (DCFH-DA) was employed for measuring of the singlet oxygen (^1^O_2_) generation capacity of PA/Ce6 nanoparticles. As illustrated in Figure 3D, after 660 nm light irradiation, the fluorescence emitted by the oxidized DCFH-DA probe in the cell was detected. Compared to free Ce6, the ROS-mediated fluorescence of PA/Ce6 nanoparticles was greater. This explained the production of ROS and proved the *in vitro* photodynamic effect of PA/Ce6 in cells. It was worth noting that after 808 nm NIR irradiation, the intracellular PA/Ce6 showed no photodynamic effect. However, when 660 nm laser and 808 nm laser were given at the same time, PA/Ce6 showed enhanced fluorescence intensity. This was due to the 808 nm laser inducing the nanoparticles to achieve the heating process and release more Ce6.

Moreover, the 1,3-diphenylisobenzofuran (DPBF) probe was used to monitor the ability of nanoparticles to generate ^1^O_2_ under illumination (Figure 3E). The DPBF probe can react with ^1^O_2_ and decompose into 1,2-dibenzoylbenzene, resulting in a reduction in its absorbance at 410 nm. It can be seen that 660 nm light can effectively excite PA/Ce6 to generate an active photodynamic effect. With the prolongation of the illumination time, the light absorption value at 410 nm decreased significantly, indicating that the nanoparticles continued to produce ROS.

### *In vitro* anti-tumor

**FIGURE 4:**
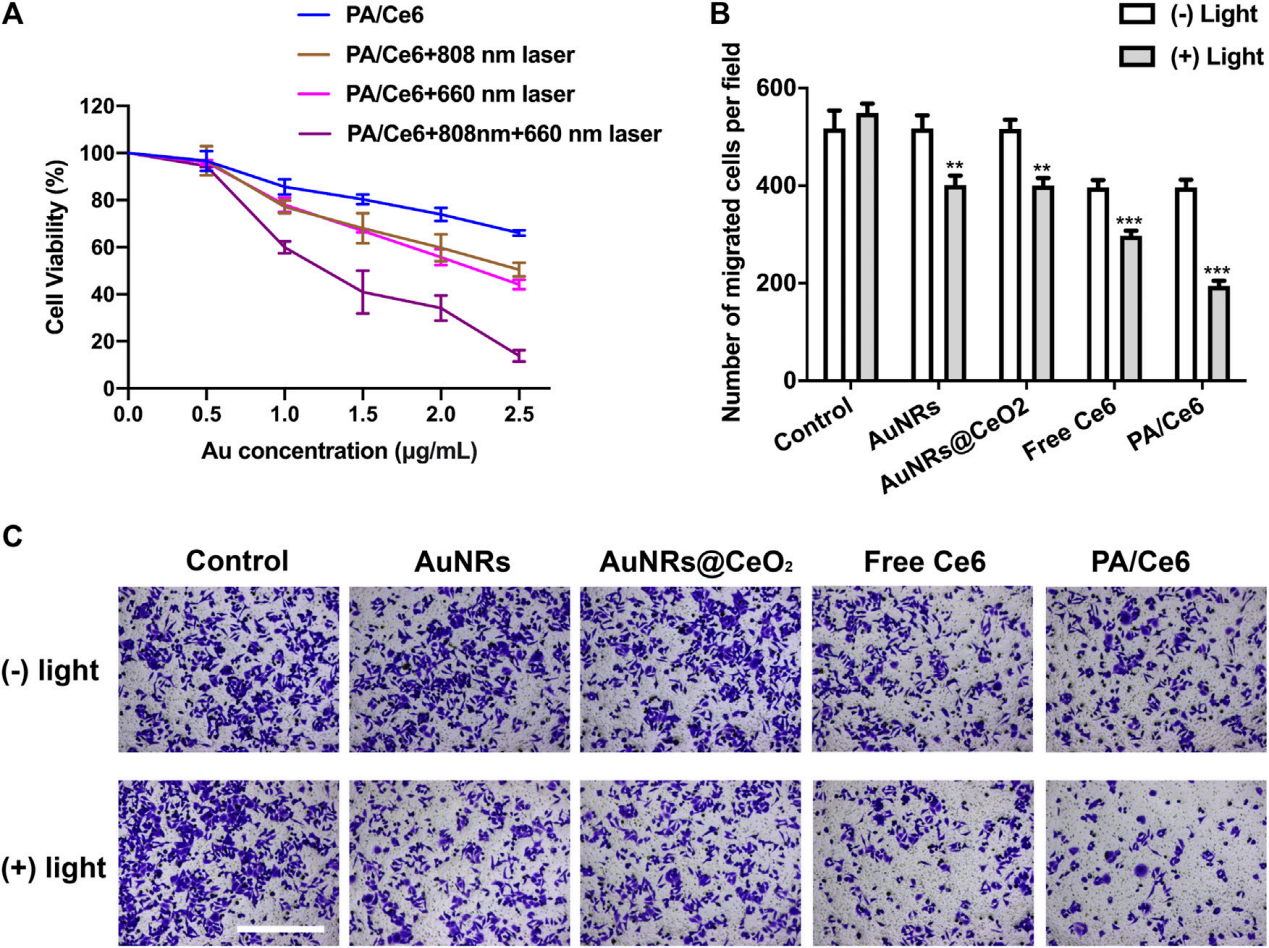
**(A)** Cell viability (OD value) of HepG2 cells treated with PA/Ce6 (with/without NIR laser irradiation) at various Au concentrations at pH 7.4. **(B,C)** Number of cells of migration after treating with AuNRs, AuNRs@CeO_2_, free Ce6, and PA/Ce6 [with/without NIR laser irradiation (LE-LS-808-XXTM/LE-LS-660-XXTM, Leoptics, Shenzhen)]. For each experiment, three independent repetitions were conducted in this study. * indicates a *p* value of <0.05; ** indicates a *p* value of <0.01.

The MTT method was conducted to detect the survival and growth of nanoparticle-treated HepG2 cells. The effect of the concentration of Au and NIR laser was studied. As shown in Figure 4A, there is a downward trend of cell survival rate with increasing Au concentration. However, at low concentrations, the cell viabilities were basically above 80%, showing good cell compatibility. In addition, after irradiating 660 and 808 nm laser, the cell viability further decreased. This was attributed to the fact that 660 nm laser induced Ce6 to generate singlet oxygen, which can effectively perish some cancer cells, while the 880 nm laser induced AuNRs to achieve photothermal conversion, leading to local heating to ablate cancer cells. As expected, after the 660 and 880 nm lasers were irradiated at the same time, the cancer cells were further inhibited, showing a good *in vitro* anti-tumor effect.

Figures 4B,C shows the migration of HepG2 cells after nanoparticle treatment. Similar to the results of MTT, NIR laser can significantly and effectively induce nanoparticles to inhibit cancer cells.

### *In vivo* anti-tumor effect

**FIGURE 5:**
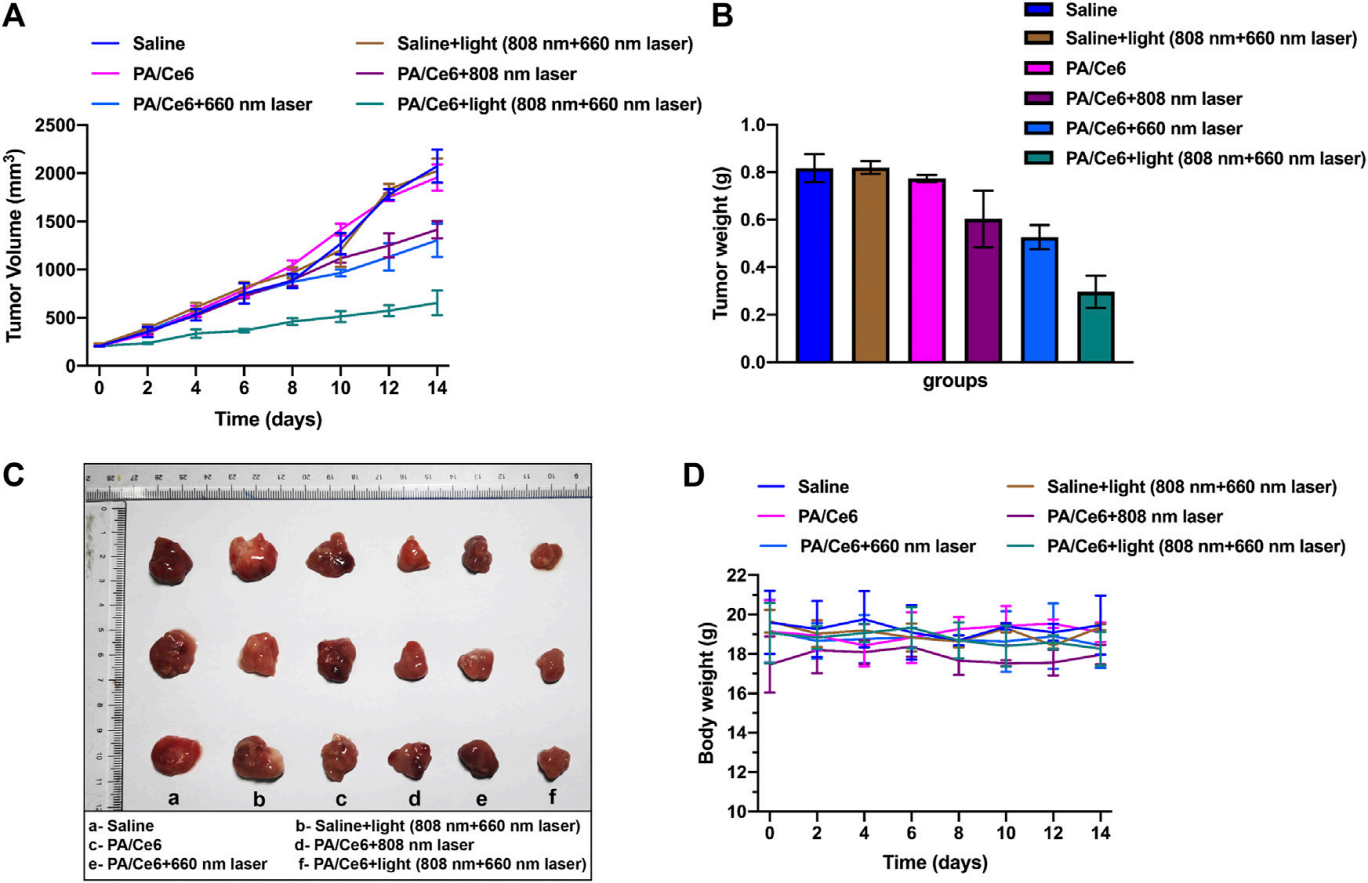
**(A)** Mouse tumor volume in different groups, **(B)** mouse tumor weight in different groups, **(C)** tumor photos of tumor-bearing mice, and **(D)** mouse body weight in different groups. For each experiment, three independent repetitions were conducted in this study. * indicates a *p* value of <0.05; ** indicates a *p* value of <0.01.

To assess the anti-tumor activity of nanoparticles *in vivo*, we studied its effect on tumor suppression in a nude mouse HCC model. The tumor model was established with HepG2 cells, and the nude mice were treated when the tumor reached to about 200 mm^3^. The effects of saline (as a control group) and NIR laser irradiation were studied. Figure 5A shows the change in tumor volume within 14 days of treatment. Consistent with the predicted results, PA-DOX showed the best tumor growth inhibitory effect under NIR laser irradiation (Figure 5B). Figure 5C showed the tumor photos of tumor-bearing mice. In addition, the physiological toxicity of nanomaterials is evaluated by monitoring the weight change of nude mice (Figure 5D). The weight of nude mice in each group remained basically stable, indicating that the nanoparticles had good biocompatibility and did not cause acute toxicity.

## Conclusion

To summarize, we have developed a novel type of nanoparticle PA/Ce6 for synergistic treatment of liver cancer. Due to the existence of AuNRs, nanoparticles had high-efficiency photothermal conversion ability to achieve PTT. In addition, it had a good controllable release effect based on temperature changes, providing a new method for the long-term release of the photosensitizer Ce6. Also, Ce6 can generate singlet oxygen under the excitation of 660 nm laser to realize PDT. The experimental results also showed that PA/Ce6 can effectively decompose hydrogen peroxide under laser irradiation, aiming to effectively alleviate the anaerobic microenvironment of tumors. In addition, in the nude mouse liver cancer model, mice treated with PA/Ce6 nanoparticles showed excellent tumor suppression and did not show acute physiological toxicity. These fruits indicate that PA/Ce6 plays a promising role for HCC treatment.

## Data Availability

The original contributions presented in the study are included in the article/Supplementary Material; further inquiries can be directed to the corresponding author.
